# Marked Hypoplasia of the Distal Phalanges in Ellis–Van Creveld Syndrome

**DOI:** 10.5334/jbsr.1552

**Published:** 2018-04-20

**Authors:** Stijn Marcelis, Geert Mortier, Filip Vanhoenacker

**Affiliations:** 1 Antwerp University Hospital, BE; 2 AZ Sint-Maarten and University (Hospital) Antwerp/Ghent, BE

**Keywords:** Ellis-van Creveld syndrome, dysplasia, radiograph

## Case report

**Figure 1:**
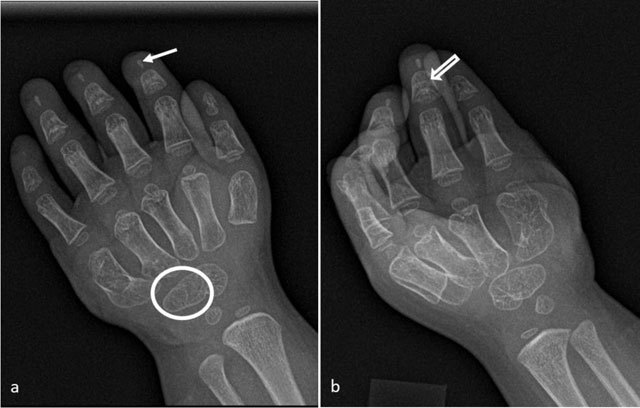
Radiographs of the left hand: **A.** Posteroanterior view showing hypoplasia of the distal phalanges (arrow at the second finger) and fusion of the hamate and capitate bones (circle). **B.** Oblique view showing cone-shaped epiphysis of the middle phalanges (open arrow at the fourth finger).

**Figure 2:**
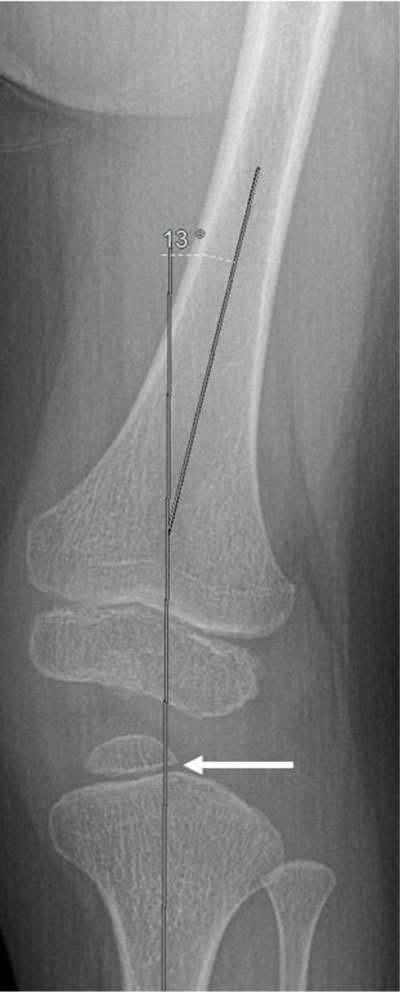
Anteroposterior radiograph of the left knee shows genu valgum (13°) and hypoplasia of the lateral proximal epiphysis of the tibia (arrow).

A newborn girl presented with a small thorax, short limbs, bilateral postaxial polydactyly with cutaneous syndactyly and dystrophic nails. Further clinical examination revealed a small indentation on the middle part of the upper lip, hypertrophic gingiva and a prominent frenulum. Neonatal radiographic evaluation showed dysplasia of multiple bones. The clinical and radiological picture was suspicious for Ellis–Van Creveld (EvC) syndrome. Therefore, a blood sample was taken for genetic investigations. The molecular analysis showed a homozygeous c.2447_2451dup(p.Val818Argfs*3) mutation of exon 14 of the EVC2 gen, confirming the diagnosis of EvC syndrome. One year later, the polydactyly was operated by the hand surgeon. Radiological follow-up revealed several additional imaging characteristics of EvC. A radiograph of the fingers showed marked hypoplasia of the distal phalanges, fusion of the hamate and capitate bones and cone-shaped middle phalanges. (Figure 1) Radiographs of the knee showed hypoplasia of the proximal tibia epiphysis, genua valga and relative shortening of the fibulae (Figure 2).

## Comment

Ellis–van Creveld syndrome (EvC), also known as chondroectodermal dysplasia, was first described in 1940. It is an autosomal recessive disorder with a prevalence around 7/1,000,000. The syndrome has several typical features: chondrodysplasia resulting in disproportionate short stature, bilateral postaxial polydactyly of the hands, ectodermal dysplasia and congenital cardiac malformations [1]. Of particular interest in our case was the marked hypoplasia and pointing of the distal phalanges and the hypoplasia of both lateral tibial epiphysis which resulted in genua valga. These skeletal anomalies may result from impaired endochondral bone formation and deficient osteoblast formation. It may also result from ectodermal dysplasia as the growth plate of the distal phalanges is in close relation to the nail matrix. Ectodermal dysplasia affects the nails and, therefore, it can also affect the distal phalanges. EvC is known to be an acromesomelic type of dwarfism, which means that the distal bones are more affected than proximal bones. The differential diagnoses include asphyxiating thoracic dystrophy (ATD), McKusick-Kaufman dysplasia (MKK), achondroplasia, Weyers acrodental dysostosis, achondroplasia and hypochondroplasia. ATD may show the same distal bone hypoplasia, but shows less nail deformities. In case of MKK, hypoplasia of the distal bones is rarely seen. Weyers acrodental dysostosis is the heterozygous manifestation of the EvC gene. It can show a similar chondroectodermal dysplasia, but heart defects, thoracic dysplasia and short stature are absent. Achondroplasia and hypochondroplasia are also examples of short-limb dwarfism but typically show a large head and no polydactyly, ectodermal dysplasia or other anomalies of the internal organs [1].

In conclusion, apart from other well-known features of EvC syndrome, marked hypoplasia of the distal phalanges is a distinctive radiological feature which should be looked for.
